# Supplementary material for: Genome wide analysis for mouth ulcers identifies associations at immune regulatory loci
Source: Nat Commun. 2019 Mar 5;10:1052. doi: 10.1038/s41467-019-08923-6 (PMC6400940; doi:10.1038/s41467-019-08923-6)
Supplement: Supplementary file 1 — Supplementary Information [file 41467_2019_8923_MOESM1_ESM.pdf]

“Genome wide analysis for mouth ulcers identifies associations at immune regulatory loci”

Dudding *et al.*

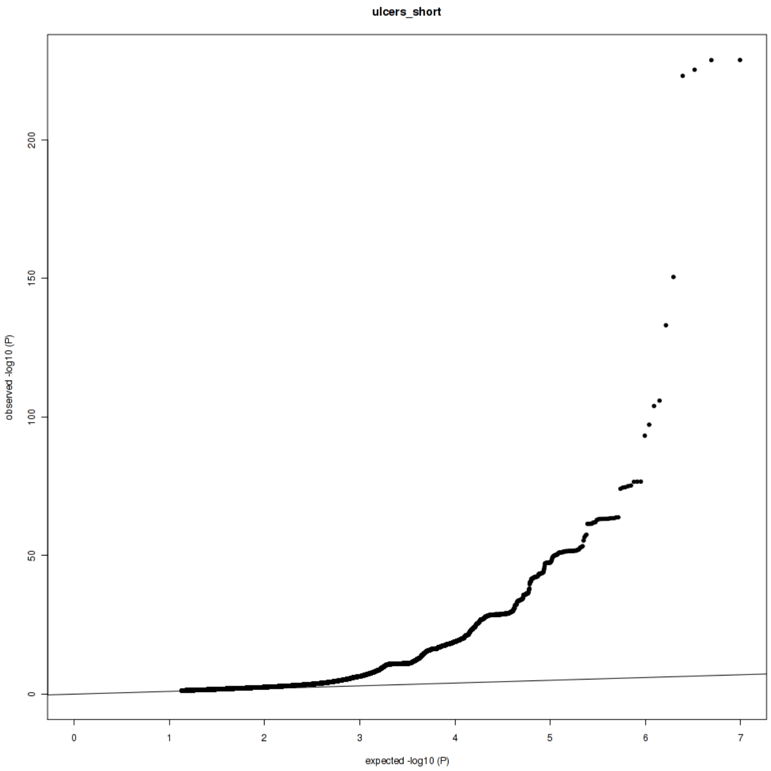

Supplementary Figure 1: QQ plot of genome-wide association analysis of self-reported ever ulcers in UK Biobank.

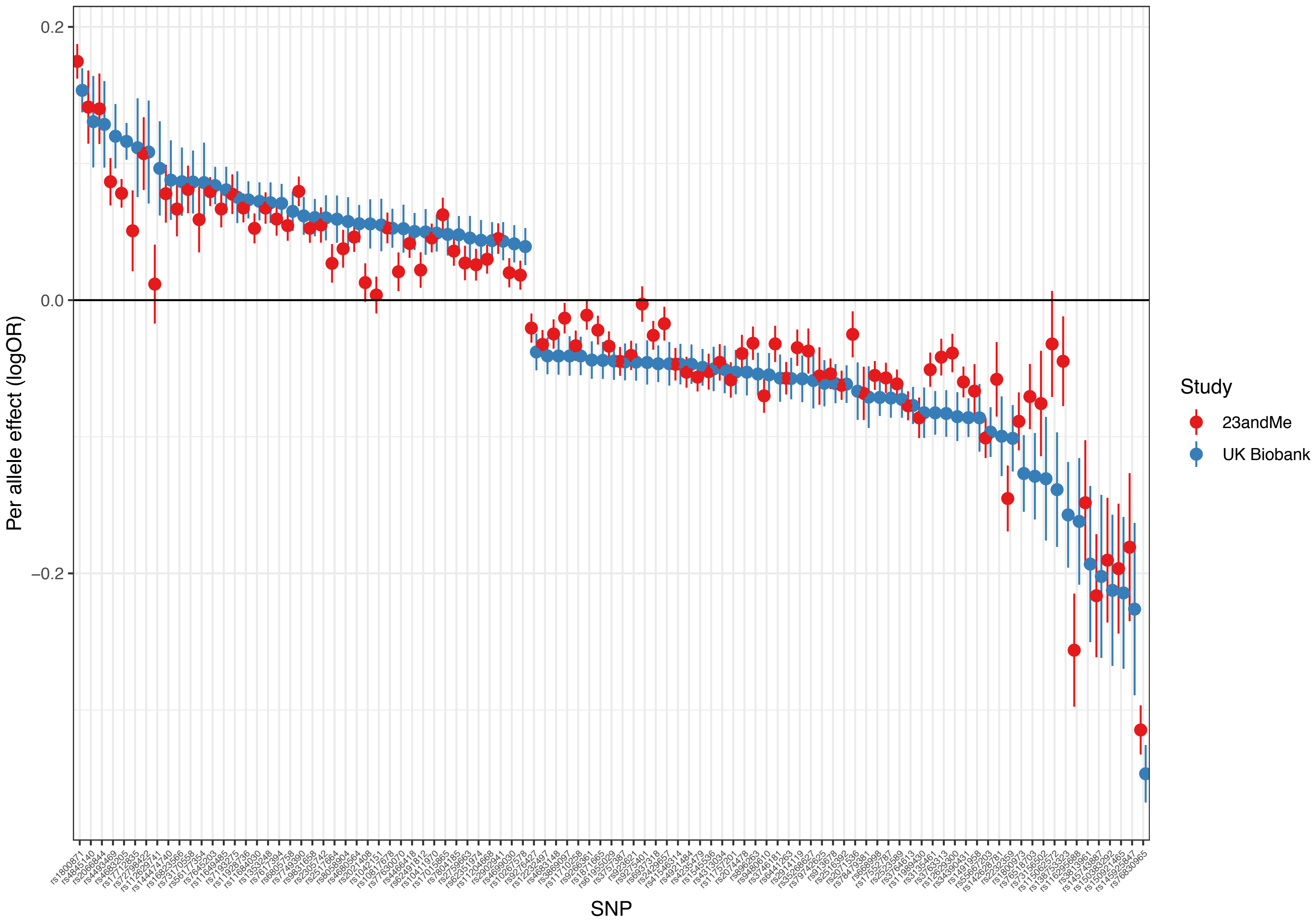

Supplementary Figure 2: Comparison of genome-wide associated variants in discovery (UK Biobank) and replication (23andMe).
Error bars indicate 95% confidence intervals.

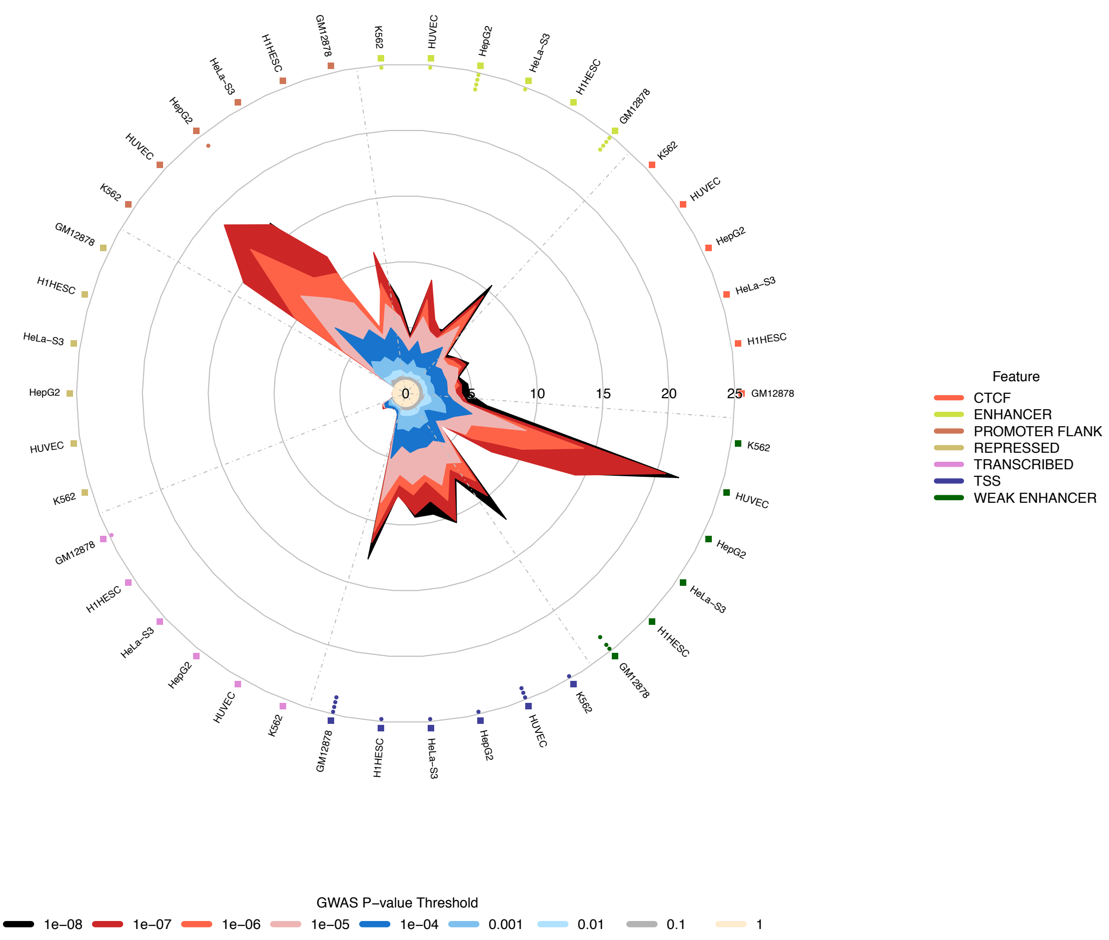

Supplementary Figure 3: Enrichment of oral ulceration variants in particular chromatin state regions.

Radial lines show fold enrichments values at eight GWAS P-value thresholds for tier 1 and 2 ENCODE cell lines. Dots in the inner ring of the outer circle denote level of evidence of enrichment (if present) at T<10-5 (outermost) to T<10-8 (innermost) and are colored with respect to the chromatin feature they test.

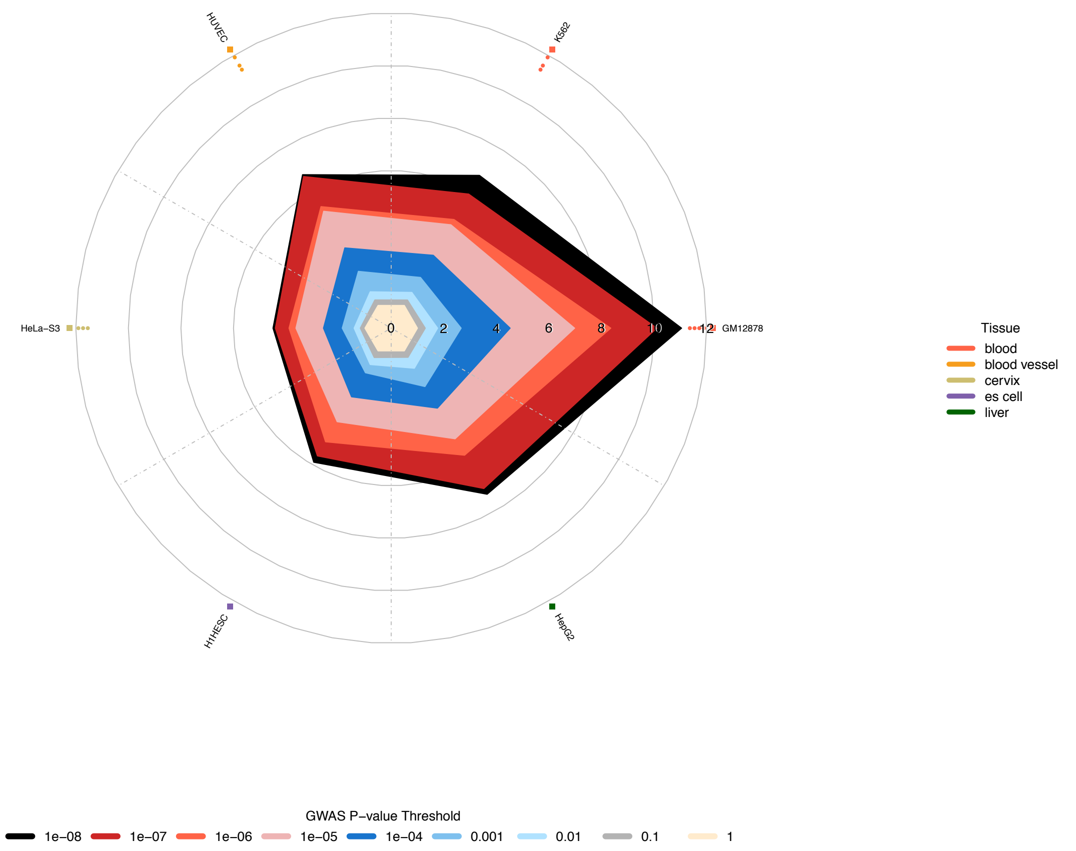

Supplementary Figure 4: Enrichment of oral ulceration variants in regions identified by FAIRE (formaldehyde-assisted isolation of regulatory elements).

Radial lines show fold enrichments values at eight GWAS P-value thresholds for tier 1 and 2 ENCODE cell lines. Dots in the inner ring of the outer circle denote level of evidence of enrichment (if present) at T<10-5 (outermost) to T<10-8 (innermost) and are colored with respect to tissue of the cell type they test.

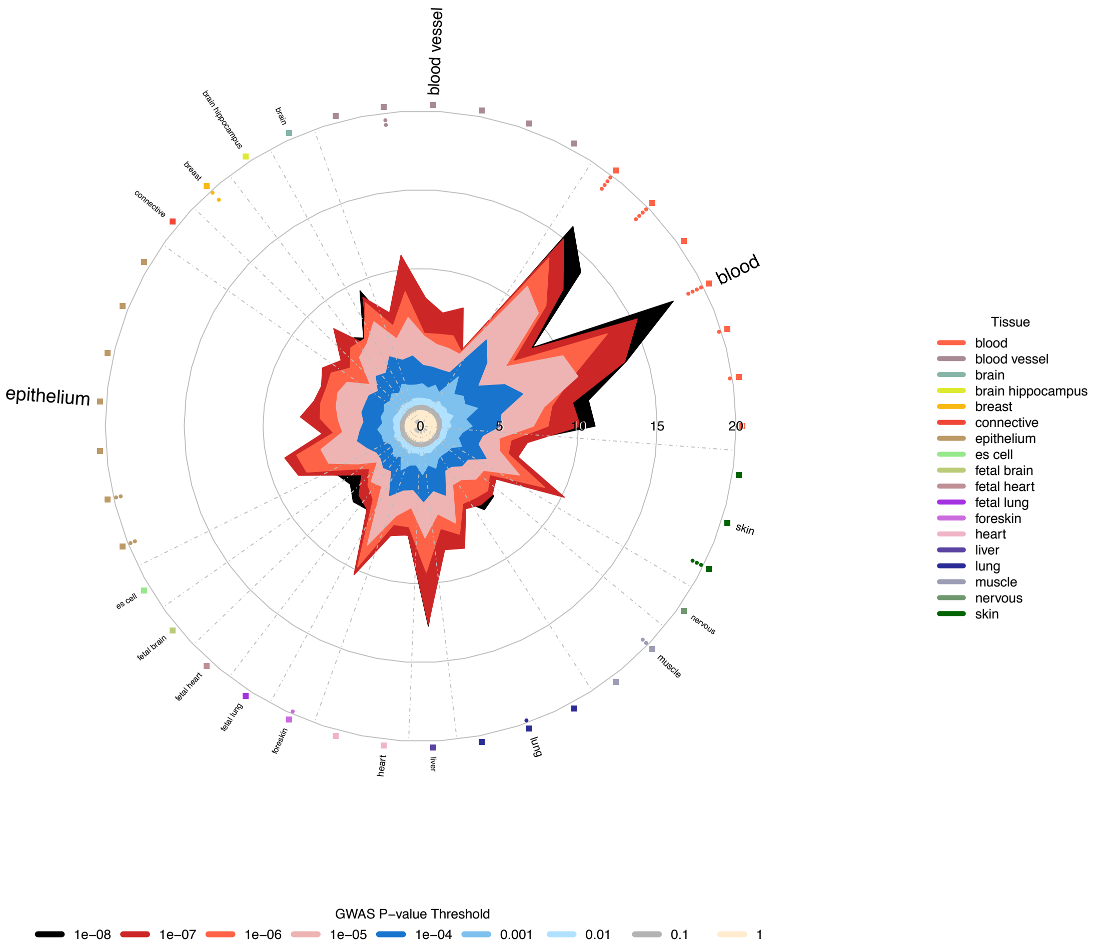

Supplementary Figure 5: Enrichment of oral ulceration variants in regions identified by DNA foot printing.

Radial lines show fold enrichments values at eight GWAS P-value thresholds for all ENCODE and Roadmap Epigenomics tissues. Dots in the inner ring of the outer circle denote level of evidence of enrichment (if present) at T<10-5 (outermost) to T<10-8 (innermost) and are colored with respect to tissue of the cell type they test.

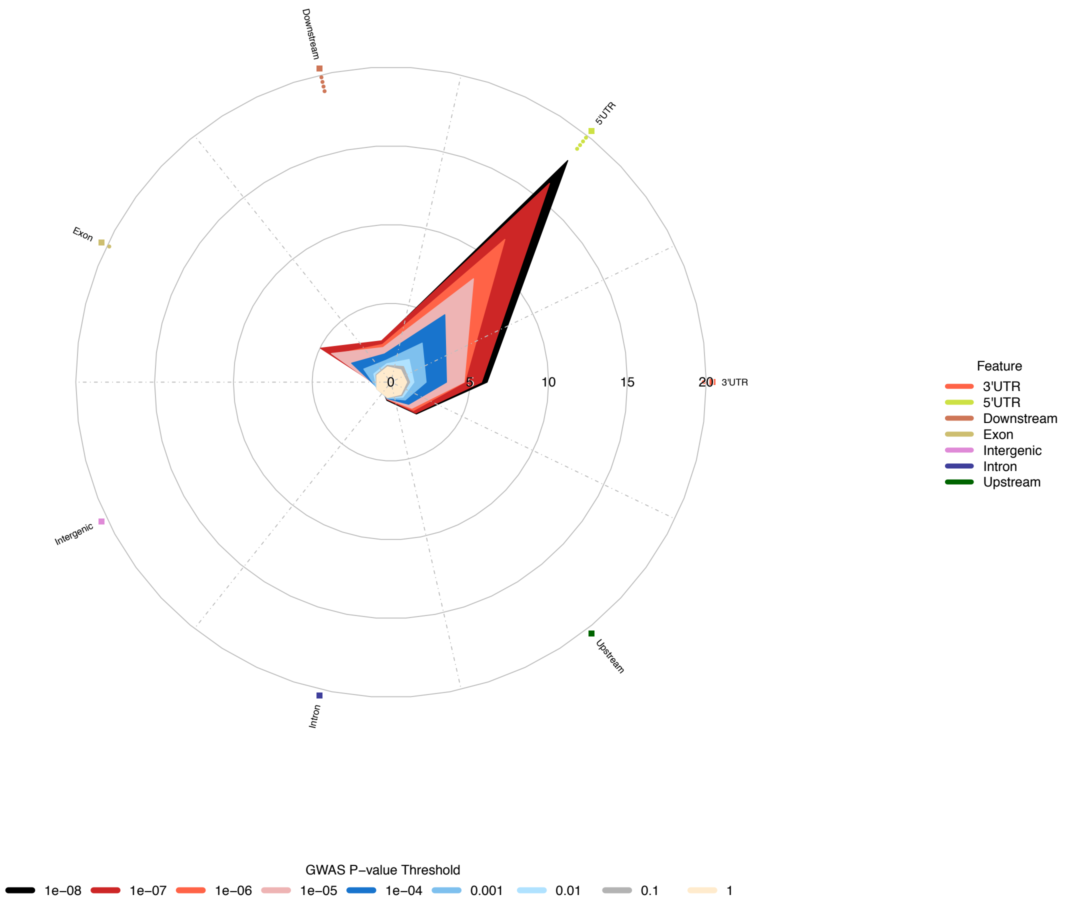

Supplementary Figure 6: Enrichment of oral ulceration variants with specific location features. Radial lines show fold enrichments values at eight GWAS P-value thresholds for all location features. Dots in the inner ring of the outer circle denote level of evidence of enrichment (if present) at T<10-5 (outermost) to T<10-8 (innermost) and are colored with respect to the location feature they test.

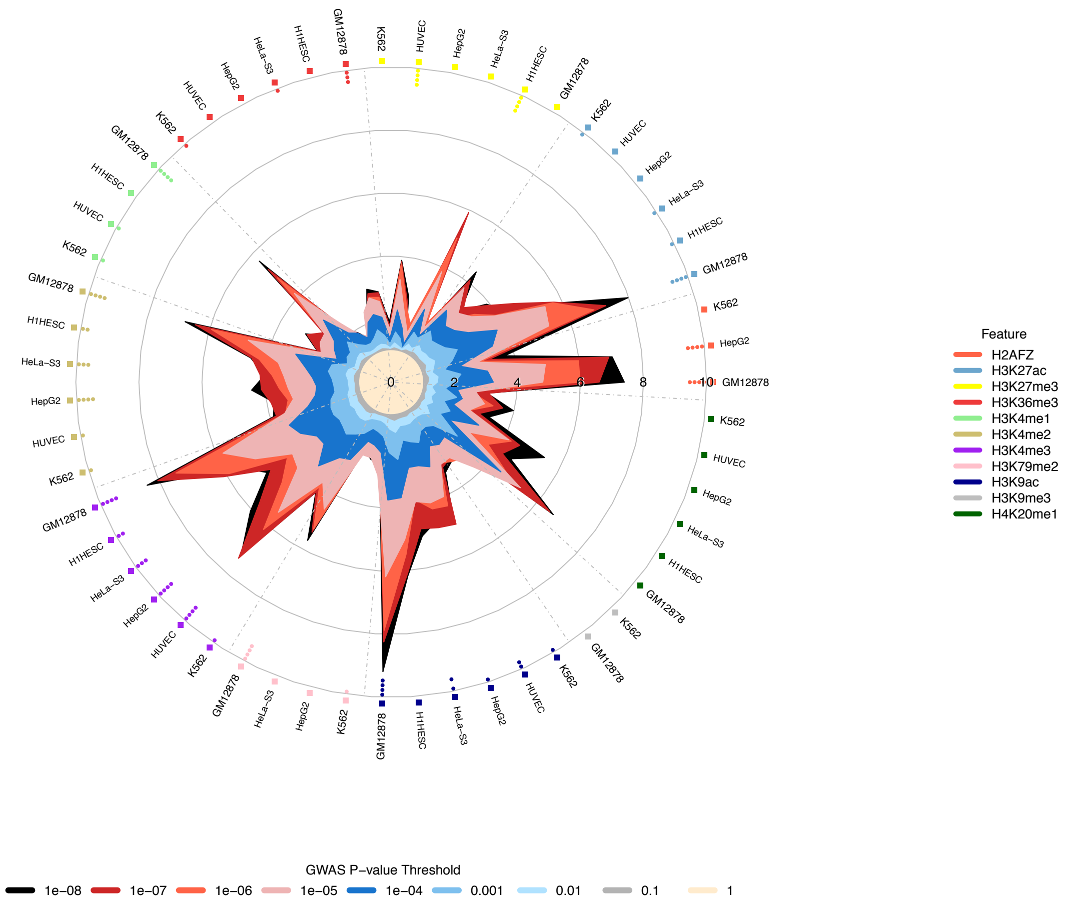

Supplementary Figure 7: Enrichment of oral ulceration variants in areas of histone modification. Radial lines show fold enrichments values at eight GWAS P-value thresholds for tier 1 and 2 ENCODE cell lines. Dots in the inner ring of the outer circle denote level of evidence of enrichment (if present) at T<10-5 (outermost) to T<10-8 (innermost) and are colored with respect to the histone modification feature they test.

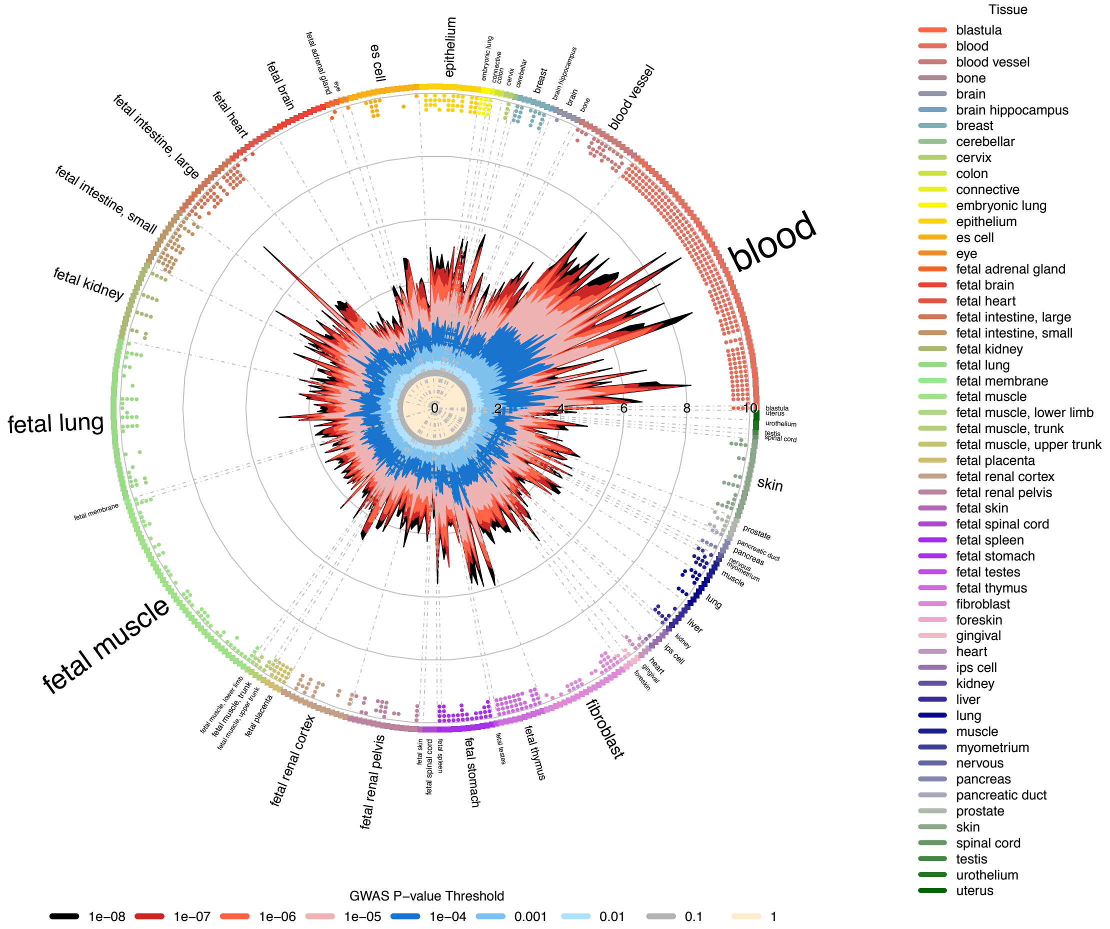

Supplementary Figure 8: Enrichment of oral ulceration variants in DNaseI Hypersensitive sites (hotspots).

Radial lines show fold enrichments values at eight GWAS P-value thresholds for all ENCODE and Roadmap Epigenomics DHS cell lines, sorted by tissue on the outer circle. Dots in the inner ring of the outer circle denote level of evidence of enrichment (if present) at T<10-5 (outermost) to T<10-8 (innermost) and are colored with respect to the tissue of the cell type they test.

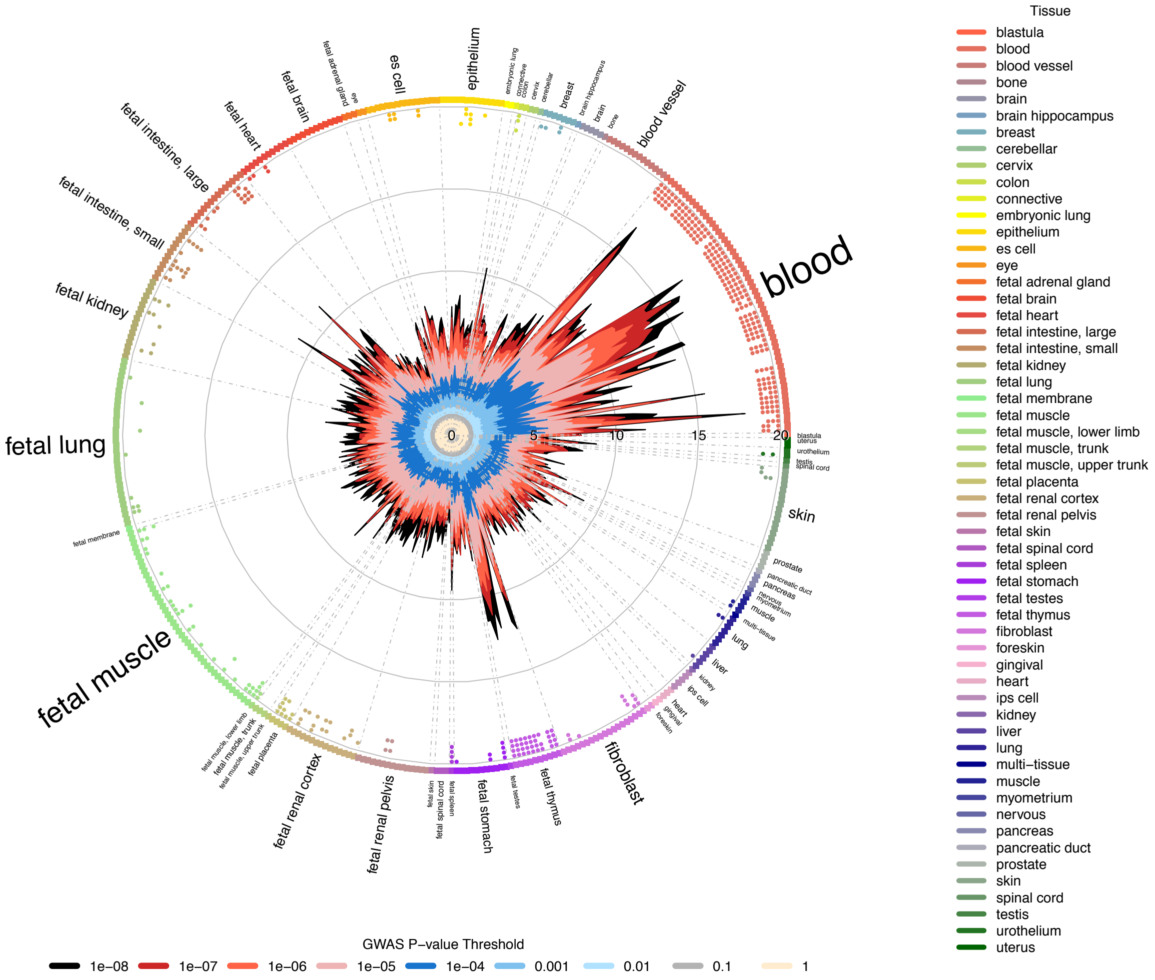

Supplementary Figure 9: Enrichment of oral ulceration variants in DNaseI Hypersensitive sites (peaks).

Radial lines show fold enrichments values at eight GWAS P-value thresholds for all ENCODE and Roadmap Epigenomics DHS cell lines, sorted by tissue on the outer circle. Dots in the inner ring of the outer circle denote level of evidence of enrichment (if present) at T<10-5 (outermost) to T<10-8 (innermost) and are colored with respect to the tissue of the cell type they test.

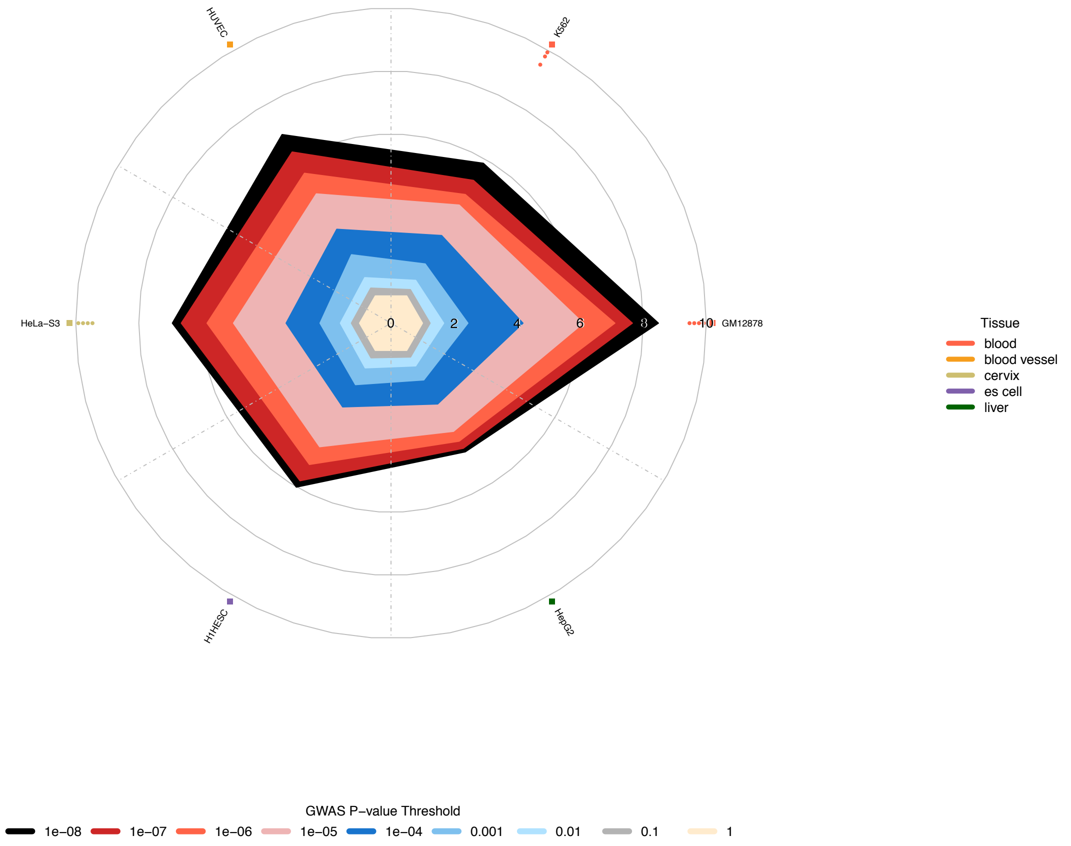

Supplementary Figure 10: Enrichment of oral ulceration variants in transcription factor binding site regions.

Radial lines show fold enrichments values at eight GWAS P-value thresholds for tier 1 and 2 ENCODE cell lines. Dots in the inner ring of the outer circle denote level of evidence of enrichment (if present) at T<10-5 (outermost) to T<10-8 (innermost) and are colored with respect to tissue of the cell type they test.

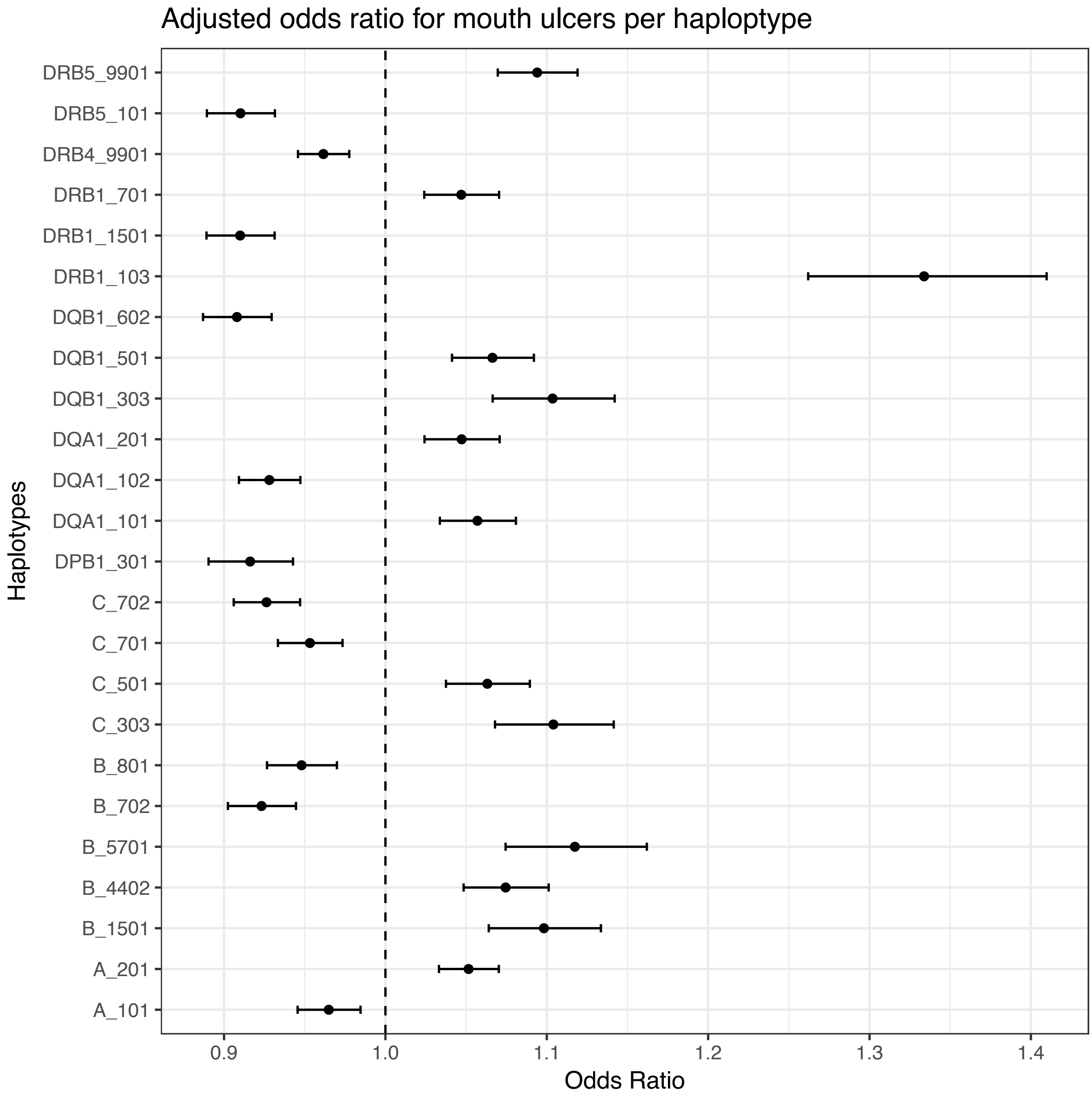

Supplementary Figure 11: Association of mouth ulcers with imputed HLA haplotypes in UK Biobank.

Results are shown for mouth ulcer associations with haplotypes passing a Bonferroni-corrected p value threshold of 0.05 and are adjusted for age, sex, genotype array and first 40 genetic principal components. Error bars indicate 95% confidence intervals.

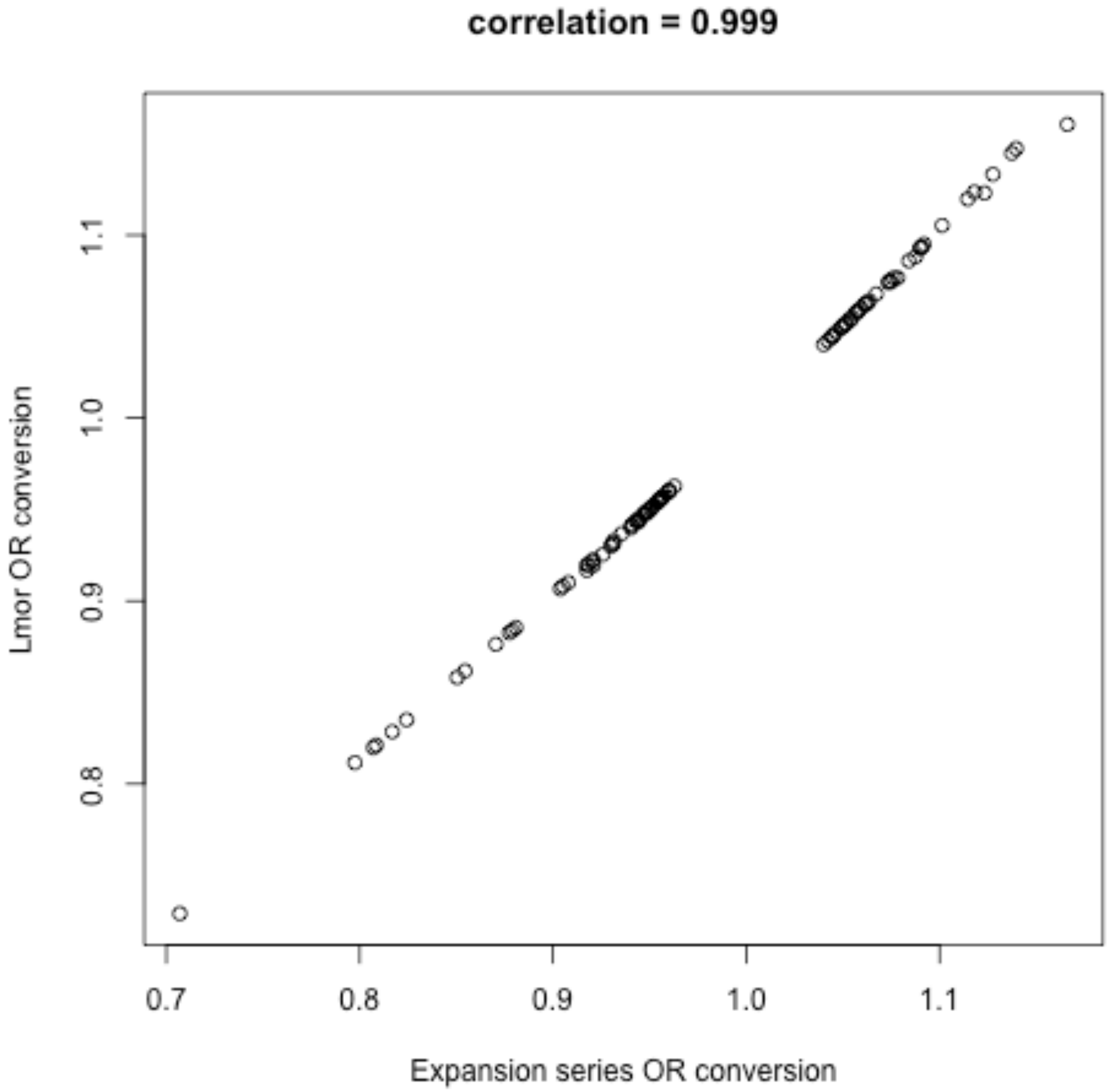

Supplementary Figure 12: Comparison of Taylor transformation expansion series OR and LMOR method that takes into account allele frequency

Correlation between OR from different methods of conversion from linear mixed model estimate.

Supplementary Table 1: HLA haplotype association adjusted analyses.

Haplotype	Odds ratio	Lower confidence interval	Upper confidence interval	P value	Haplotype frequency in cases	Haplotype frequency in controls
DRB1_103	1.334	1.262	1.410	2.03E-24	0.022	0.017
DQB1_602	0.908	0.887	0.930	6.45E-16	0.132	0.144
DRB1_1501	0.910	0.889	0.931	1.61E-15	0.134	0.146
DRB5_101	0.910	0.889	0.932	1.65E-15	0.144	0.146
DRB5_9901	1.094	1.070	1.119	6.17E-15	0.857	0.845
DQA1_102	0.928	0.909	0.947	9.94E-13	0.180	0.192
B_702	0.923	0.902	0.945	7.35E-12	0.139	0.149
C_702	0.926	0.906	0.947	1.37E-11	0.149	0.159
DPB1_301	0.916	0.890	0.943	1.85E-09	0.095	0.101
C_303	1.104	1.068	1.141	5.66E-09	0.061	0.056
B_1501	1.098	1.064	1.134	6.38E-09	0.069	0.063
B_4402	1.074	1.048	1.101	9.31E-09	0.119	0.112
DQB1_303	1.104	1.066	1.142	1.70E-08	0.057	0.052
A_201	1.052	1.033	1.070	2.29E-08	0.284	0.274
B_5701	1.117	1.074	1.162	2.74E-08	0.043	0.039
DQB1_501	1.066	1.041	1.092	1.21E-07	0.127	0.120
C_501	1.063	1.038	1.089	8.90E-07	0.119	0.113
DQA1_101	1.057	1.034	1.081	1.08E-06	0.148	0.141
DRB4_9901	0.962	0.946	0.978	3.36E-06	0.641	0.650
B_801	0.948	0.927	0.970	4.70E-06	0.139	0.145
C_701	0.953	0.933	0.973	7.77E-06	0.170	0.177
DQA1_201	1.047	1.024	1.071	4.71E-05	0.150	0.144
DRB1_701	1.047	1.024	1.070	4.80E-05	0.150	0.145
A_101	0.965	0.946	0.985	5.30E-04	0.189	0.195

Supplementary Table 2: Polygenic risk score for mouth ulcers predicting mouth ulcers case status.

	TW and AG study combined: Adults and adolescents (n=3,562)				TW study: adolescents (n=1,572)				TW and AG study combined: adults (n=1,990)			
PRS threshold	Beta	SE	P	R2	Beta	SE	P	R2	Beta	SE	P	R2
0.00000005	0.0219	0.0082	0.0079	0.00205	0.0368	0.0115	0.0014	0.0058	0.0159	0.01	0.1131	0.0011
0.00001	0.0224	0.0082	0.0066	0.00214	0.0366	0.0114	0.0014	0.0057	0.0137	0.0101	0.1725	0.0008
0.001	0.0294	0.0082	0.0003	0.0037	0.0363	0.0113	0.0013	0.0056	0.0234	0.0101	0.0213	0.0023
0.01	0.0221	0.008	0.0059	0.00209	0.0233	0.0111	0.0359	0.0023	0.0313	0.01	0.0018	0.0042
0.05	0.0098	0.008	0.223	0.00041	0.0144	0.0109	0.1859	0.0009	0.0154	0.0101	0.1282	0.001
0.1	0.0085	0.008	0.2881	0.00031	0.0123	0.0109	0.2623	0.0006	0.0173	0.01	0.0846	0.0013
0.5	0.0035	0.008	0.6593	0.000053	0.0049	0.011	0.6529	0.0001	0.015	0.01	0.133	0.001
1	0.0047	0.008	0.552	0.000096	0.0062	0.011	0.5689	0.0002	0.0163	0.01	0.1039	0.0011

All models include the following covariates: age, sex, principal components 1-4. P=p-value, PRS=polygenic risk score, R2=variance explained by PRS.

Supplementary Table 3: Polygenic risk score for mouth ulcers predicting mouth ulcer severity.

	TW study: combined adults/adolescents (n=2,442)				TW study: adolescents (n=1,572)				TW study: adults (n=870)			
PRS threshold	Beta	SE	P	R2	Beta	SE	P	R2	Beta	SE	P	R2
0.00000005	0.0235	0.0065	0.0003	0.0073	0.0259	0.0083	0.0018	0.0088	0.0272	0.0093	0.0033	0.0032
0.00001	0.025	0.0065	0.00012	0.0082	0.0264	0.0082	0.0014	0.0092	0.0298	0.0093	0.0015	0.0038
0.001	0.0255	0.0064	0.000063	0.0086	0.0237	0.0081	0.0036	0.0074	0.0412	0.0092	0.000008	0.0073
0.01	0.0225	0.0062	0.00029	0.0067	0.018	0.008	0.0246	0.0042	0.0411	0.0091	0.0000065	0.0072
0.05	0.0185	0.0062	0.0027	0.0045	0.0154	0.0078	0.0501	0.0031	0.0317	0.0092	0.00063	0.0043
0.1	0.0164	0.0061	0.0074	0.0036	0.0127	0.0079	0.1071	0.0021	0.0318	0.009	0.00046	0.0043
0.5	0.0131	0.0062	0.0328	0.0023	0.0098	0.0079	0.2129	0.0013	0.0255	0.0091	0.0053	0.0028
1	0.0134	0.0061	0.0296	0.0024	0.0101	0.0079	0.1993	0.0014	0.0259	0.0091	0.0046	0.0029

All models include the following covariates: age, sex, principal components 1-4. P=p-value, PRS=polygenic risk score, R2=variance explained by PRS.

Supplementary Table 4: Drug repurposing

Drug	Target	Disease	Max phase	Molecule type
INFLIXIMAB	TNF	rheumatoid arthritis	Phase IV	Antibody
COLLAGENASE CLOSTRIDIUM HISTOLYTICUM	COL11A2	Dupuytren Contracture	Phase IV	Enzyme
BORTEZOMIB	PSMB8	multiple myeloma	Phase IV	Small molecule
ETANERCEPT	TNF	rheumatoid arthritis	Phase IV	Protein
OXYBATE	GABBR1	obstructive sleep apnea	Phase IV	Small molecule
ADALIMUMAB	TNF	rheumatoid arthritis	Phase IV	Antibody
USTEKINUMAB	IL12A	immune system disease	Phase IV	Antibody
MARAVIROC	CCR5	HIV infection	Phase IV	Small molecule
GOLIMUMAB	TNF	ankylosing spondylitis	Phase IV	Antibody
CERTOLIZUMAB PEGOL	TNF	rheumatoid arthritis	Phase IV	Antibody
CARFILZOMIB	PSMB8	multiple myeloma	Phase IV	Protein
IXAZOMIB CITRATE	PSMB8	multiple myeloma	Phase IV	Small molecule
BACLOFEN	GABBR1	alcohol dependence	Phase IV	Small molecule
OCRIPLASMIN	COL11A2	macular holes	Phase IV	Enzyme
BRIAKINUMAB	IL12A	immune system disease	Phase III	Antibody
ONERCEPT	TNF	psoriatic arthritis	Phase III	Protein
ARBACLOFEN PLACARBIL	GABBR1	multiple sclerosis	Phase III	Small molecule
CENICRIVIROC	CCR5	non-alcoholic steatohepatitis	Phase III	Small molecule
VICRIVIROC	CCR5	AIDS	Phase III	Small molecule
ARBACLOFEN	GABBR1	autism spectrum disorder	Phase III	Small molecule
APLAVIROC	CCR5	HIV infection	Phase III	Small molecule
OMECAMTIV MECARBIL	MYL3	heart failure	Phase III	Small molecule
MARIZOMIB	PSMB8	glioblastoma multiforme	Phase III	Small molecule
PEXACERFONT	CRHR1	irritable bowel syndrome	Phase III	Small molecule
PF-04634817	CCR5	diabetic nephropathy	Phase II	Small molecule
EMICERFONT	CRHR1	irritable bowel syndrome	Phase II	Small molecule
PRO-140	CCR5	HIV infection	Phase II	Antibody
AZD2423	CCR2	pain	Phase II	Small molecule
LESOGABERAN	GABBR1	gastroesophageal reflux disease	Phase II	Small molecule
BAMINERCEPT	LTA	rheumatoid arthritis	Phase II	Protein
APOLIZUMAB	HLA-DRB5	lymphoma	Phase II	Antibody
IPH-2101	KIR2DL3	multiple myeloma	Phase II	Antibody
MLN-1202	CCR2	diabetic nephropathy	Phase II	Antibody
PEGSUNERCEPT	TNF	rheumatoid arthritis	Phase II	Protein
VERUCERFONT	CRHR1	irritable bowel syndrome	Phase II	Small molecule
CCX140	CCR2	diabetic nephropathy	Phase II	Small molecule
PATECLIZUMAB	LTA	rheumatoid arthritis	Phase II	Antibody
LIRILUMAB	KIR2DL3	head and neck squamous cell carcinoma	Phase II	Antibody
AZD4818	CCR1	chronic obstructive pulmonary disease	Phase II	Small molecule

OZORALIZUMAB	TNF	rheumatoid arthritis	Phase II	Antibody
PLOVAMER ACETATE	HLA-DRB1	relapsing-remitting multiple sclerosis	Phase II	Small molecule
CCX354	CCR1	rheumatoid arthritis	Phase II	Small molecule
AZD5672	CCR5	rheumatoid arthritis	Phase II	Small molecule
PLACULUMAB	TNF	rheumatoid arthritis	Phase II	Antibody
SSR125543	CRHR1	unipolar depression	Phase II	Small molecule
ONO-2333MS	CRHR1	unipolar depression	Phase II	Small molecule
INCB-9471	CCR5	HIV infection	Phase II	Small molecule
SCH-708980	IL10	Leishmaniasis	Phase II	Antibody
OPROZOMIB	PSMB8	multiple myeloma	Phase I	Small molecule
CCR5MAB004	CCR5	HIV infection	Phase I	Antibody
ASG-5ME	SLC44A4	prostate carcinoma	Phase I	Antibody
