# Supplementary material for: Genome wide analysis for mouth ulcers identifies associations at immune regulatory loci
Source: Nat Commun. 2019 Mar 5;10:1052. doi: 10.1038/s41467-019-08923-6 (PMC6400940; doi:10.1038/s41467-019-08923-6)
Supplement: Supplementary file 3 — Description of Additional Supplementary Files [file 41467_2019_8923_MOESM3_ESM.docx]

**Description of Additional Supplementary Files**

File Name: SupplementaryData1

Description: Summary statistics for 97 lead variants in all studies.

File Name: SupplementaryData2

Description: Gene prioritization analysis in DEPICT

File Name: SupplementaryData3

Description: Gene set enrichment analysis in DEPICT

File Name: SupplementaryData4

Description: Tissue enrichment analysis in DEPICT

File Name: SupplementaryData5

Description: Genetic correlation analysis in LD Hub

File Name: SupplementaryDate6

Description: rhoHESS local genetic correlation between mouth ulcers and neuroticism

File Name: SupplementaryDate7

Description: rhoHESS local genetic correlation between mouth ulcers and depressive symptoms
